# Perioperative Nutritional Aspects in Total Pancreatectomy: A Comprehensive Review of the Literature

**DOI:** 10.3390/nu13061765

**Published:** 2021-05-22

**Authors:** Niccolò Furbetta, Annalisa Comandatore, Desirée Gianardi, Matteo Palmeri, Gregorio Di Franco, Simone Guadagni, Giovanni Caprili, Matteo Bianchini, Lorenzo Maria Fatucchi, Martina Picchi, Luca Bastiani, Giandomenico Biancofiore, Giulio Di Candio, Luca Morelli

**Affiliations:** 1 General Surgery Unit, Department of Translational Research and New Technologies in Medicine and Surgery, University of Pisa, Via Paradisa 2, 56124 Pisa, Italy; n.furbetta@hotmail.it (N.F.); a.comandatore@libero.it (A.C.); gianardi.d@gmail.com (D.G.); palmeri.matteo@gmail.com (M.P.); gregoriodifranco@gmail.com (G.D.F.); simone5c@virgilio.it (S.G.); mabcap@libero.it (G.C.); bianchini.matteo@yahoo.it (M.B.); lorenzofatucchi@gmail.com (L.M.F.); martinapicchi@yahoo.it (M.P.); giuliodicandio@gmail.com (G.D.C.); 2 Institute of Clinical Physiology, National Council of Research, 56124 Pisa, Italy; luca.bastiani@ifc.cnr.it; 3 Division of Transplant Anesthesia and Critical Care, University of Pisa, 56124 Pisa, Italy; g.biancofiore@med.unipi.it

**Keywords:** total pancreatectomy, total pancreatectomy with islet auto-transplantation, nutrition, nutritional status, nutritional support

## Abstract

Total pancreatectomy (TP) is a highly invasive procedure often performed in patients affected by anorexia, malabsorption, cachexia, and malnutrition, which are risk factors for bad surgical outcome and even may cause enhanced toxicity to chemo-radiotherapy. The role of nutritional therapies and the association between nutritional aspects and the outcome of patients who have undergone TP is described in some studies. The aim of this comprehensive review is to summarize the available recent evidence about the influence of nutritional factors in TP. Preoperative nutritional and metabolic assessment, but also intra-operative and post-operative nutritional therapies and their consequences, are analyzed in order to identify the aspects that can influence the outcome of patients undergoing TP. The results of this review show that preoperative nutritional status, sarcopenia, BMI and serum albumin are prognostic factors both in TP for pancreatic cancer to support chemotherapy, prevent recurrence and prolong survival, and in TP with islet auto-transplantation for chronic pancreatitis to improve postoperative glycemic control and obtain better outcomes. When it is possible, enteral nutrition is always preferable to parenteral nutrition, with the aim to prevent or reduce cachexia. Nowadays, the nutritional consequences of TP, including diabetes control, are improved and become more manageable.

## 1. Introduction

Indications for total pancreatectomy (TP) decreased over time and today are limited to a few cases of advanced pancreatic cancer, diffuse Intraductal Papillary Mucinous Neoplasm (IPMN), pancreatic metastasis or chronic pancreatitis [1,2,3,4].

Patients affected by pancreatic cancer, a very aggressive gastrointestinal malignancy, often present cachexia, vomiting and malabsorption, which contribute to an impairment of both performance status and quality of life, representing risk factors for bad surgical outcomes and can cause enhanced toxicity to chemo-radiotherapy [5,6,7]. Patients affected by chronic pancreatitis also present malnutrition and a high rate of postoperative complications [8,9,10].

TP is a highly invasive procedure proposed with or without simultaneous auto-islet transplantation [11] for some types of chronic pancreatitis and also for the treatment of locally advanced pancreatic cancer. TP with islet auto-transplantation (TPIAT) can be considered in patients with severe chronic pancreatitis with irreversible pancreas injury which leads to malabsorption, weight loss and muscle decrease [12,13].

Surgical consequences of TP include intestinal denervation, loss of gastric pacemaker (because of the removal of interstitial cells of Cajal) causing diabetes mellitus, intestinal disordered function and delayed gastric emptying. Furthermore, extensive lymphadenectomy causes the absence of the inhibition of sympathetic nerves which induce diarrhea and alterations of the physiological intestinal homeostasis until severe malnutrition. Hyperglycemia interferes with leukocyte function, influencing granulocyte adherence, phagocytosis and chemotaxis, causing a depressed bactericidal capacity [14,15].

In malnourished patients undergoing TP, nutritional factors and perioperative nutritional therapy are considered to be important to improve clinical outcomes, including tolerance to chemotherapy [16]. Some studies tried to analyze the benefits of nutritional therapy and the association between nutritional aspects and the outcome of patients undergoing TP.

A comprehensive review was conducted to summarize the available recent evidence about the influence of nutritional factors in TP and to evaluate the association between nutritional therapies and postoperative outcomes in patients undergoing TP.

## 2. Materials and Methods

### 2.1. Search Strategy

A systematic literature search was carried out in PubMed for studies published between 1 January 2010 and 10 October 2020 to identify studies addressing the nutritional status of patients undergoing TP, as well as the nutritional therapies before and after TP. The search was limited to articles in English. Conference abstracts, case series with a number of TP < 10 and case reports were excluded. The references of the studies were also reviewed and included. Keywords included were: total pancreatectomy, diet therapy, immunonutrition, enteric feeding, parenteral nutrition, enteral nutrition, symbiotic agents, enhanced recovery after surgery and islet transplantation and supplementation.

### 2.2. Selection Criteria, Data Extraction and Quality Assessment

After removing duplicate records, abstracts were screened independently by two investigators (AC and NF) to determine eligible studies for further analysis. Full-text articles of the remaining records were subsequently retrieved and reviewed. All discrepancies and disagreements were resolved through consensus. We included in our analysis manuscripts focusing on the nutritional status or metabolic assessment of patients undergoing TP and those focusing on perioperative management and post-operative nutritional therapies in order to identify the aspects that can influence the outcome of patients undergoing TP. Relevant data were extracted from each included report. The information extracted comprised authors, publication year, study design, number of patients/studies included, outcomes and conclusions. The present study was conducted according to the Preferred Reporting Items for Systematic Reviewers and Meta-Analyses (PRISMA) guidelines [17].

## 3. Results

### 3.1. Literature Research

**Figure 1:**
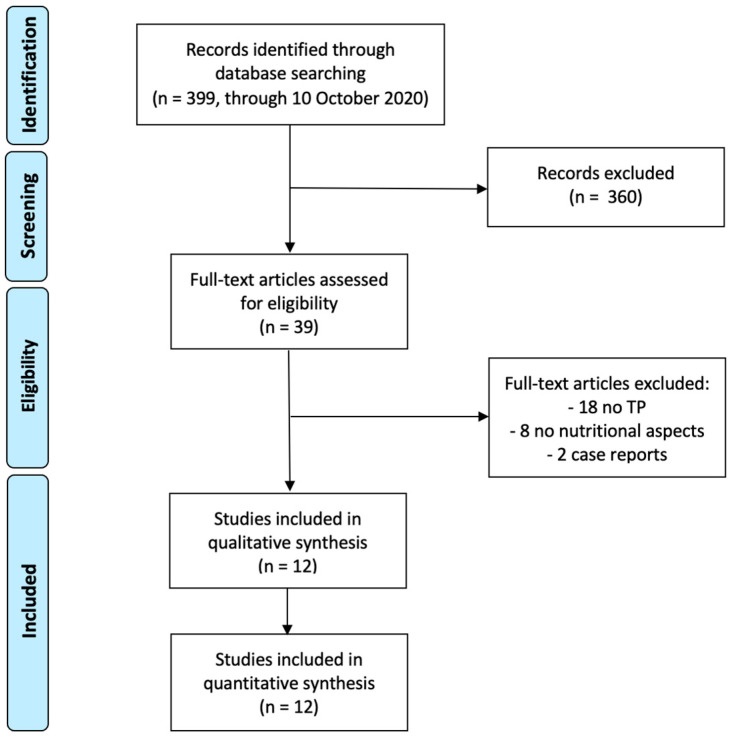
PRISMA diagram of literature research.

Figure 1 shows the systematic searching process. We identified 399 articles from PubMed (MEDLINE). Three hundred and sixty studies were excluded based on the abstracts’ review. After reviewing the remaining full-text articles, a further 27 of 39 selected studies were omitted because of the following reasons: 18 reported about gastrointestinal surgery in general or pancreatectomies, but with few or 0 cases of TP; 8 articles did not analyze nutritional aspects and 2 were case reports.

### 3.2. Study Characteristics

Data extracted from the 12 finally selected articles are reported in Table 1. Five of the twelve articles analyzed perioperative nutritional aspects after TP and 7/12 after TPIAT. The topics highlighted in the selected articles are analyzed in the discussion section, enriched with other studies from the literature, as follows: pre-operative nutritional status; peri-operative nutritional support and nutritional consequences of TP, including diabetes and glycemic control; glucagon; fatty liver and hepatic steatosis; fatty pancreas and diabetes and enteroendocrine hormones.

Moreover, 3 articles addressed nutritional status, including 1 also discussing preoperative sarcopenia, 1 analyzing the preoperative BMI of a pancreatic donor, and 1 highlighting the importance of glycemic control and nutritional status after TP. Nutritional support was examined in 3 studies. Postoperative nutritional status and consequences after TP or TPIAT were analyzed in 5 studies, including 3 studies addressing quality of life and endocrine or exocrine insufficiency, 1 addressing hepatic steatosis and 1 addressing the enteroendocrine system.

## 4. Discussion

### 4.1. Pre-Operative Nutritional Status

The prognostic nutritional index (PNI), developed by Smale et al. in 1981 [30], was calculated from the serum albumin, triceps skin-fold thickness, serum transferrin and delayed hypersensitivity reaction, and reflects patients’ nutritional and immune status. In 1984, Onodera et al. [31] simplified this index into the currently used formula calculated by summing the serum albumin concentration with five times the lymphocyte count in the peripheral blood. Thus, a low PNI indicates malnutrition, reduced immunity and neutrophil function [32].

Several Authors [33,34,35,36] described the leading role of the PNI as a prognostic factor. Vashi et al. [33] documented that, in pancreatic cancer patients, improvement in nutritional status during cancer treatment decreases the risk of mortality independently of sex, previous treatment history and evidence of biological anticancer activity. Geng et al. [34] confirmed the role of the PNI in predicting survival in patients affected by advanced pancreatic cancer.

The retrospective study published by Kanda et al. [35], which included 268 patients who had undergone pancreatic resection (25 TP) for adenocarcinoma of the pancreas, analyzed the predictive value of preoperative nutritional status for postoperative outcomes (survival, complications). In multivariate analysis, a preoperative low PNI resulted to be an independent prognostic factor for poor survival, identifying a cut-off value of 45. Furthermore, low preoperative albumin concentration and PNI were significantly associated with postoperative complications. The authors concluded that the PNI is associated with overall survival and postoperative complications, in particular pancreatic fistula.

Ikeguchi et al. [37] tried to analyze the clinical importance of preoperative and postoperative PNI in patients with pancreatic ductal adenocarcinoma undergoing surgery. They analyzed 50 patients with PDAC who had undergone curative resection, of whom two were total pancreatectomies. Patients were divided into two groups: 23 patients with a PNI at 2 months postoperatively that had been recovered to the preoperative level, and the remaining 27 patients with a PNI at 2 months postoperatively that had not reached the preoperative level. The overall median survival time in the first group was significantly longer than that in the latter group. The multivariate overall analysis demonstrated that a good recovery of the postoperative PNI was strongly correlated with a better prognosis. The authors concluded that the improvement of the nutritional status before and after surgery may have improved the postoperative prognosis in patients with PDAC.

Nanashima et al. [38] retrospectively evaluated the relationship between the PNI and clinical–pathological factors, surgical data and postoperative morbidity in 323 patients who had undergone pancreatic resections (the procedure was not specified). They concluded that the PNI was not associated with pancreatic architecture, known to be associated with postoperative complications. The PNI correlated with preoperative metabolic parameters and postoperative protein and albumin levels but was not different in the presence or absence of each complication. Nanashima et al. suggested the necessity of a modified formula specific to pancreatic surgery.

Total pancreatectomy with islet auto-transplantation (TPIAT) can be considered in patients with severe chronic pancreatitis with irreversible pancreas injury which leads to malabsorption, weight loss and muscle decrease [12,13]. Reaching a high transplanted islet cell mass predicts long- and short-term islet graft function and insulin independence [20,39,40].

Metabolic assessment could be useful to address the optimal timing for TPIAT in chronic pancreatitis. In fact, the optimal timing for TPIAT can be difficult to determine, considering the preservation of islet mass without proceeding to surgery too early in the disease course. Lundberg et al. [19] tried to find better preoperative predictors of islet yield. In fact, even if the role of MRI in the prediction of islet yield is analyzed [41], the real potential islet yield is currently unknown until pancreatectomy is performed [40,42]. The relationship between measures from frequent sample IV glucose-tolerance tests (FSIVGTT) and mixed-meal tolerance tests (MMTT) and islet mass was examined. Data on 60 non-diabetic patients with chronic pancreatitis who underwent TPIAT were retrospectively analyzed to identify predictors of islet mass <2500 IEQ/kg. The authors found a weak relationship between stimulated insulin, C-peptide levels and glycemic measures with the isolated islet mass in adult patients with chronic pancreatitis undergoing TPIAT. On the other hand, they found that a stimulated C-peptide and normal fasting glucose could help to identify the risk for low islet yield, thus more severe postoperative diabetes.

Cachexia, vomiting, malnutrition and malabsorption, which characterize both chronic pancreatitis and pancreatic cancer [5,6,7,8,22], play a key role in the pathogenesis of sarcopenia [43]. Sarcopenia is the decreasing of skeletal muscle mass and represents a parameter for patients’ reserves. Patients with chronic pancreatitis gradually lose endocrine and exocrine function involving malabsorption and undernutrition [44,45].

The association of these factors with the parallel attendance of alcohol abuse in some patients brings to muscle exhaustion and fat-soluble vitamin decrease [20]. In a recent consensus paper of the International Study Group on Pancreatic Surgery [46], sarcopenia is considered as a relevant factor for short- and long-term outcomes after pancreatic resection, prolonging duration in hospital stay and significantly rising long-term mortality. A recent prospective study by Olesen [47], including 182 patients with chronic pancreatitis, showed that sarcopenia was present in 17%. The relationship between sarcopenia and an increased risk of hospitalization, increased number of in-hospital days and reduced survival were documented. However, sarcopenia’s effects on the outcome of patients that had undergone TPIAT for chronic pancreatitis are less known.

Trikudanathan et al. [20] analyzed results obtained in 138 patients who had undergone TPIAT. The CT imaging-based Skeletal Muscle Index (SMI) was applied to CT scans performed 6 months before surgery to evaluate sarcopenia. Patients were divided into three islet dose categories: <2500 IEQ/kg, 2500–5000 IEQ/kg, >5000 IEQ/kg and were evaluated one year after TPIAT. In this study, 33% of patients had sarcopenia. There were no significant differences in the incidence of complications in the sarcopenia and non-sarcopenia groups. However, more patients with sarcopenia are required to be discharged to residential rehabilitation. Moreover, significantly higher levels of HbA1c and lower basal and stimulated C-peptide were observed in the sarcopenic group after 1 year. Indeed, 84% of patients with sarcopenia required insulin, while the percentage of patients without sarcopenia who required insulin was 68%. Patients with pre-operative sarcopenia had also a poor islet yield at 1-year follow-up, and this data is maybe related to modified glucose distribution caused by a decreased muscle mass in sarcopenic patients. According to this study, a pre-operative evaluation is fundamental, together with the optimization of pre-operative muscle mass with an exercise regimen and nutritional support with the administration of protein, vitamin D, antioxidant nutrients and long-chain polyunsaturated fatty acids.

Body Mass Index (BMI) is proven to be a poor tool for assessing the nutritional status and disease prognosis in pancreatic cancer [35,48]. However, a retrospective study published by Takita et al. [21] evaluated the BMI of pancreatic donors as a predictor after TPIAT. This study demonstrated that the high BMI group had better islet isolation and better autologous islet cell transplantation outcomes while the low BMI group had a higher score in pancreatitis. Lower BMI is also related to fibrotic and inflammatory changes of the pancreas, which are the main causes of lower islet yield in these patients. In the low BMI group, malnutrition can be considered as one of the reasons for poor islet yield. The authors observed a larger size of islets in the low BMI group than in the high BMI group, with a smaller final production volume per pancreas in the low BMI group in respect to the high BMI one. This difference was related to supposed suffering due to a chronic inflammatory environment. They also observed decreased C-peptide levels in the low BMI group, while the high BMI group presented better long-term graft function and islet yields.

Furthermore, in patients with chronic pancreatitis, decreased BMI and sarcopenia may lead to decreased functional capacity, which may have an impact on the quality of life [47,49].

Some authors reported that albuminemia reflects nutritional status and is independently associated with postoperative complications. In fact, hypoalbuminemia is related to postoperative morbidity and mortality as well as surgical site infection [35,50,51,52,53,54].

In a recently published retrospective article, Nakano et al. [55] investigated the clinicopathological features and prognostic factors associated with pre- and postoperative serum albumin levels in 196 patients with curatively resected PDAC. The surgical procedures included were pancreaticoduodenectomy, distal pancreatectomy and TP. The study found that serum albumin level at post-operative month 12 was an independent prognostic factor both for disease-free and overall survival, defining serum albumin as a potential biomarker to predict the prognosis of patients who have undergone curative pancreatectomy for PDAC.

Cytokines could also play a role in cachexia [56,57], as pro-inflammatory cytokines interfere in cachexia mechanisms so that the inhibition of these cytokines could improve BMI by reducing weight loss in pancreatic cancer [22,58].

In conclusion, the studies analyzed showed the importance of nutritional status in patients undergoing TP. Indeed, in those undergoing TP for PDAC, an optimal nutritional status can improve prognosis, while in those undergoing TPIAT for chronic pancreatitis, a preoperative metabolic assessment, including sarcopenia and BMI, could be useful to predict islet mass and thus identify patients with a high risk of severe postoperative diabetes.

### 4.2. Peri-Operative Nutritional Support

Perioperative nutrition represents an important factor that influences clinical outcomes such as survival rate and chemotherapy response in patients who have undergone TP. The primary aim of parenteral or enteral nutrition in patients who are candidates to TP is to prevent or reduce cachexia. In fact, a preoperative low prognostic nutrition index is associated with poor survival and a higher postoperative complications rate [35].

Karagianni et al. [22] published in 2012 a review of the literature addressing the nutritional support before and after pancreatectomy for pancreatic cancer and chronic pancreatitis. The review demonstrated no benefits in using parenteral nutrition, which is even associated with a higher rate of complications. According to the European Society for Clinical Nutrition and Metabolism guidelines on clinical nutrition, in acute and chronic pancreatitis [10], parenteral nutrition may be indicated only in patients with gastric outlet obstruction and in those with a complex fistulation disease, or in cases of patients unable to consume food or exhibiting an intolerance of enteral nutrition.

A prospective randomized study conducted by Brennan et al. in 1994 [59] had already demonstrated that routine postoperative parenteral nutrition after major pancreatic surgery is not recommended. In fact, the results of the study showed no benefit in using adjuvant parenteral nutrition in patients who had undergone major pancreatic resection for malignancies, while complications were significantly greater, in particular those associated with infection.

A Danish retrospective study published by Andersen et al. [23] enrolled 97 patients who had undergone TP between 2009 and 2014 and compared postoperative parenteral nutrition with protocolled insulin treatment to intravenous glucose treatment. The parenteral nutrition cohort (n = 57) had an infusion rate of more than 20 h with SmofKabiven since the first postoperative day, the carbohydrate per day was body-weight-dependent and for every 10 g of carbohydrate 1 international unit (IU) of insulin (Novorapid) was administered. The glucose cohort (n = 40) received a continuous isotonic glucose 5% infusion (60 mL/h), using 2 IU of insulin every 10 g carbohydrate until oral nutrition was re-established. Both groups received supplementary doses of insulin if plasma glucose values went over 10 mmol/L. Complications and outcome measures were observed during the first 13 post-operative days. Plasma glucose control data demonstrated that parenteral nutrition with insulin treatment per protocol makes better glycemic control if compared to glucose infusion. In the parenteral nutrition group, a decreased non-infectious postoperative complications rate was registered, although no increase of hypoglycemia was observed. A decreased prevalence of sepsis was found due to complete nutrition with carbohydrate, protein and lipids for the first 8 days after surgery. These results are in contrast with a previous review which documented an increased risk of infectious complications associated with parenteral nutrition [60]. However, these results are hardly comparable with those of the previous review because it considered partial pancreatectomy, where endogenous glucagon and insulin production is maintained. This study also demonstrated that sufficient oral feeding is impossible to obtain within 7 days after surgery.

Wagar et al. [24] analyzed 67 patients that underwent TPIAT who were divided into two cohorts: a group was intraoperatively managed with standard fluid therapy (SFT), while a second group was managed with a goal-directed fluid therapy (GDFT). What emerged was that the GDFT group required less intraoperative transfusions and less intraoperative fluid resuscitation with a comparable 30-day complications rate and 1-year islet graft function in respect to the SFT cohort.

A randomized, controlled trial published by Liu et al., focusing on gastrointestinal oncologic surgery [61], tried to determine the clinical efficiency and safety of hormone therapy combined with hypocaloric parenteral nutrition in patients with gastrointestinal cancer. The authors measured hormones and protein metabolites, immune function, clinical outcome and adverse events in 100 patients with a nutrition risk screening score of 3 or 4 that were undergoing surgery for gastrointestinal cancer. They described a therapeutical nutritional regime characterized by a short-term administration of recombinant growth hormone, octreotide, insulin and hypocaloric parenteral nutritional which increase protein synthesis and immune function, reducing postoperative infectious complications.

The role of enteral nutrition has been analyzed by a large number of studies, mainly that in decreasing complications rate in patients undergoing pancreatoduodenectomy for cancer [22,62]. According to these studies, relevant parameters such as enteral formula could change clinical outcomes, leading to the importance of immunonutrition with arginine and omega-3-fatty acids if compared to standard enteral nutrition and parenteral nutrition. Indeed, enteral nutrition enriched by medium-chain triglycerides and protein is demonstrated to increase prealbumin and protein plasma levels, resulting in a reduced length of hospitalization when compared with isocaloric protein enteral immunonutrition [63]. The key role of eicosapentaenoic acid in an oral diet was also documented and how the administration of fish oil [64] can improve metabolic and nutritional status in patients with pancreatic carcinoma was also described.

Comparing enteral and parenteral nutrition has been the object of several trials. A review by Liu et al. [65] supported that enteral nutrition was better than parenteral nutrition with regard to clinical and biochemical parameters. This work included a prospective randomized clinical trial published by Braga et al. [66], including 257 patients with stomach, pancreas or esophagus cancer. Patients were randomized to receive postoperative total parenteral nutrition or early enteral nutrition. The nutritional goal was reached in 100/126 (79.3%) patients in the early enteral nutrition group and 128/131 (97.7%) patients in the total parenteral nutrition group. The alteration of serum electrolyte levels was significantly lower in the early enteral nutrition group compared to the parenteral nutrition one, while the overall complications rate was similar with no differences for either infectious and noninfectious complications, length of hospital stay and mortality. Furthermore, the costs were compared, concluding that enteral nutrition was less expensive than parenteral nutrition.

Since TP causes exocrine insufficiency, it was demonstrated that an effective pancreatic enzyme therapy should be supported by routine calcium and vitamin supplementation, calorie-dense meals, Vitamin D and selenium supplementation [22,67].

An experimental study [68] performed on dogs subjected to TP analyzed the role of fructose infusion to obtain a glucagon decrease after hepatic glucose uptake. Since fructose stimulates liver glucose uptake, they hypothesize the possibility to limit stress-induced hyperglycemia in fructose-supported patients. Although net hepatic glucose uptake increased after fructose infusion, this experimental study demonstrated that fructose had short-lived beneficial effects on hepatic glucose uptake and cannot be used as a therapy to treat long-term stress-induced hyperglycemia.

The use of postoperative albumin supplementation is widely debated in the literature. Some studies demonstrated that early postoperative albumin supplementation does not improve clinical outcomes [22,69]. A prospective randomized study including 127 hypoalbuminemic patients who had undergone gastrointestinal surgery [69] investigated postoperative hypoalbuminemia, nutritional status, postoperative fluid balance, postoperative complications and postoperative hospital stay. The study concluded that albumin administration in the early stage of postoperative hypoalbuminemia is not beneficial in correcting hypoalbuminemia or in improving clinical outcomes.

On the contrary, Nakano et al. [55] underlined the importance of the recovery rate of serum albumin at 12 postoperative months as a potential biomarker for predicting the prognosis of patients with curatively resected PDAC.

### 4.3. Nutritional Consequences of TP

TP is associated with postoperative morbidity and metabolic consequences resulting in lifelong insulin-dependent diabetes, with the risk of severe hypoglycemia and a substantial impact on quality of life (QoL) [70,71]. However, advances in surgical techniques and glycemic monitoring, and the development of synthetic insulin and pancreatic enzymes for postoperative treatment could improve outcomes and increase indications for TP.

#### 4.3.1. Diabetes and Glycemic Control

In the 1930s, Woodyatt described the combination of insulin sensitivity and hypoglycemic unawareness as “brittle” diabetes [72]. Pancreatogenic diabetes following TP complicates post-surgical management and negatively influences QoL. Pancreatogenic diabetes is characterized by the absence of the major glucoregulatory hormones insulin and glucagon and instability and frequent hypoglycemia [73,74], and its management is crucial. Some authors underlined its complexity [70] whereas others concluded that it could be well-managed, similarly to type 1 DM [1,71,75,76,77].

A systematic review published by Scholten et al. [25], including 1536 patients who had undergone TP, revealed a substantial rate of diabetes-related morbidity, but nevertheless acceptable and stabilized levels of HbA1c in the first year after TP, indicating reasonable management, particularly in the more recent studies. Indeed, the authors concluded that diabetes treatment after TP has recently improved, making it more manageable.

A retrospective cohort study published by Shi et al. in 2017 [18] analyzed 52 patients from 2007 to 2015 that had undergone TP, in which continuous intravenous insulin infusion was administered from the third postoperative day. For glycemic control, two variables were considered: fasting blood glucose (FBG) and HbA1c. The variables considered for determining nutritional status were BMI, serum total protein, albumin, prealbumin and the prognostic nutritional index (PNI: albumin g/L + 5 × total lymphocyte count × 10^9^/L). FBG and HbA1c levels increased at the third month but returned to preoperative levels at 12 months; BMI showed a non-stop decrease; serum total protein and albumin levels increased up to 12 months after surgery, while serum prealbumin maintained a lower level even at 12 months. What they observed through a univariate analysis was that 16 patients (69.6%) of 23 with higher early postoperative FBG had postoperative complications while only 34.5% of 29 patients with early postoperative FBG (less than 155 mg/dL) had complications. A number of 60.9% of patients belonging to the group with prealbumin postoperative levels less than 155 mg/dL had complications, compared to 27.6% of the group with higher prealbumin postoperative levels. The multivariate analysis demonstrated that high early postoperative FBG and low early prealbumin levels were significantly associated with postoperative complications. This study also analyzed tumor recurrence with univariate analysis, detecting that patients with high FBG preoperative levels (>110 mg/dL), venous invasion by tumor, HbA1c postoperative levels up to 7%, and postoperative PNI less than 45 had a decreased recurrence-free survival. Additionally, the multivariate analysis demonstrated that these factors were significant and independent risk factors for tumor recurrence. Factors such as preoperative DM and high FBG, high postoperative HbA1c, low postoperative serum total protein, albumin and prealbumin were associated with adverse prognosis. Indeed, overall survival in multivariate analysis was much poorer in patients with HbA1c postoperative levels up to 7% when compared to patients with HbA1c levels <7%. Regarding BMI, the study revealed that alcohol history was related to lower postoperative BMI. Therefore, what total pancreatectomy determines is breakage of glycemic and nutritional status balance, where glycemic control can bring to a new metabolic assessment, thus preventing complications, recurrence and improving survival. The authors concluded that recovering nutritional status and improving glycemic control after TP are important to prevent early complications and tumor recurrence and therefore to improve survival. Glycemic management and nutritional support enable the achievement of these goals for at least 3 months after TP.

Concerning TPIAT, a recent study [78] demonstrated that after TPIAT patients developed hypoglycemia symptoms after consuming a meal rich in carbohydrates, roux-en-Y choledocho-jejunostomy and gastro/duodeno-jejunostomy, sometimes included in TPIAT, play a role in nutrients absorption and hypoglycemia.

#### 4.3.2. Glucagon

In literature, it is well known that after TP, glucagon secretion is absent or strongly reduced [79,80,81]. It is also widely demonstrated that maintaining a blood insulin/glucagon ratio level similar to the physiological level can significantly improve nutritional metabolic status after TP [82].

In normal conditions, hypoglycemia stimulates glucagon secretion to increase blood glucose levels with glycogenolysis. A recent study by Bogachus et al. [26] analyzed data of 10 patients who had undergone TPIAT following their history of hypoglycemia. They hypothesized that intrahepatic transplanted islets were enclosed by high levels of glucose released by glycogenolysis that could inhibit the glucagon response to hypoglycemia. Patients were compared with 10 age-, sex-, and body-mass-index-matched control subjects, and the only differences observed were lower insulin and C-peptide and higher fasting glucose levels in patients who had undergone TPIAT in respect to control ones, while glucagon levels remained unchanged. In conclusion, initially high glucose levels followed by hypoglycemia with an absent glucagon response is a mechanistic sequence that contributes to postprandial hypoglycemia after TPIAT.

The pancreas is primarily responsible for maintaining euglycemia even during exercise. Under normal physiological conditions, reciprocal changes in insulin and glucagon secretion, epinephrine and the autonomic nervous system in response to exercise allow euglycemia to be maintained [83,84,85]. When the blood glucose concentration begins to decrease due to tissue utilization, insulin secretion is decreased and glucagon stimulates liver glycogenolysis to release glucose which enters the systemic circulation. Bogachus et al. [86] sought to establish how intrahepatically transplanted islets may inaccurately regulate insulin and glucagon secretion in relation to systemic glucose levels in patients undergoing TPIAT during physical exercises. In fact, these islets could be dysregulated as they are surrounded by free glucose during glycogenolysis [78]. They analyzed patients undergoing TPIAT and a control group who performed an aerobic capacity test on a bicycle. They hypothesized that abnormalities in hepatic glucose production may be a contributing factor to post-exercise hypoglycemia. They compared 14 patients who had undergone TPIAT and 10 control subjects matched for age and BMI, analyzing blood samples to measure glucose metabolism and counterregulatory hormones. This study identified a deficiency in endogenous glucose production as a potential contributing mechanism to hypoglycemic episodes during exercise in patients who had undergone TPIAT.

The review published by Karagianni et al. [22] underlines the presence of an exceeding hyperglycemic response to glucagon after TP, suggesting the development of increased sensitivity to exogenous glucagon. Indeed, the metabolic response to glucagon is considerably more pronounced after TP than in Type I diabetic patients [87], suggesting that glucagon responsiveness is enhanced in the chronic hormone-deficient state. However, in these patients, hypoglycemia is frequently seen, and this can be caused by misregulated hepatic glucose production. Heptulla et al. [88] suggested the role of glucagon rescue injection in preventing late postprandial hypoglycemia in patients with Type I diabetes.

#### 4.3.3. Fatty Liver and Hepatic Steatosis

After TP, it is possible to observe the development of lipid storage in the liver, even if not so frequently. This phenomenon, also described in experimentally pancreatectomized animals, seems to be related to weight loss, malabsorption and uncontrolled diabetes [22,74].

The occurrence of hepatic steatosis after pancreatic surgery is known to be related to the remnant pancreatic function and nutritional status [89,90]. Hata et al. [27] analyzed results in 43 patients undergoing TP in order to identify other risk factors in addition to the remnant pancreatic function and to clarify the association between postoperative hepatic steatosis and pancreatic exocrine insufficiency. Sixteen of forty-three patients developed hepatic steatosis after TP, with significant deterioration in controlling nutritional status score and BMI. The development of hepatic steatosis was correlated with female sex, early postoperative serum albumin levels and pancreatic exocrine insufficiency at multiple linear regression analysis. On the other hand, high-dose pancreatic enzyme replacement therapy may reduce hepatic steatosis occurring after pancreatectomy.

#### 4.3.4. Fatty Pancreas and Diabetes after Tpiat

A retrospective single-center cohort study published by Kizilgul et al. [28] hypothesized a pathogenic mechanism in Type 3c diabetes where intrapancreatic fat (in chronic pancreatitis and diabetes) in pancreatic tissue biopsies play a role in diabetes development after TPIAT. Indeed, intrapancreatic fat determinates fatty acids release, triglycerides storage, oxidative stress and proinflammatory factors response [91,92,93,94]. Additionally, pancreatic fat deposition could indicate insulin resistance, as it seems to be related to the presence of non-alcoholic fatty liver disease and metabolic syndrome [95,96]. The patients enrolled in this study were divided into two cohorts: low pancreatic fat (n = 53) and high pancreatic fat (n = 26). One year after surgery, only one patient of the 24 with high-fat pancreases was insulin-independent (4%), against 17 (32.7%) of the 53 patients with low-fat pancreases. Moreover, full insulin dependence was common in high-fat pancreas patients (70.8% vs. 46.2% in the low-fat pancreas). This study suggests poorer diabetes outcomes after TPIAT in the high pancreas fat group, suggesting that intrapancreatic fat might lead to beta-cell dysfunction, even if mechanisms are still largely unknown. A limit of this study was the disregard of other confounding factors because of the wide complexity and variability of the disease.

#### 4.3.5. Enteroendocrine Hormones after Tpiat

The enteroendocrine system [97] is composed of a complex interplay of the gastrointestinal tract and pancreatic hormones. This system regulates a great number of functional properties of the gut, including motility and coordination of insulin secretion in response to nutrients ingestion. Glucagon-like peptide 1 (GLP-1) and peptide YY (PYY), secreted by the L cells [98] after a meal in response to a nutrient load promote satiety and reduce gastric and intestinal motility [99]. Furthermore, GLP-1 augments glucose-dependent insulin secretion and β-cell growth [100,101,102,103].

McEachron et al. [29], in a very recent study, analyzed a homogeneous cohort group of 34 patients who underwent TPIAT between 2010 and 2013 to evaluate the possible changes in enteroendocrine hormones after TPIAT. Patients did not have diabetes before surgery and HbA1c levels were <6%. They were randomized in sitagliptin or placebo for 12 months after surgery and they were evaluated after a mixed-meal tolerance test (MMTT) before surgery and after 12 and 18 months in order to test pancreatic islet production. For the first time, they identified alterations before and after surgery of incretin hormones such as GLP-1 and PYY and the islet hormone PP. GLP-1 and PYY values after stimulation were higher after TPIAT, maybe because of the anatomical changes after surgery, while PP levels were decreased but without statistical significance. This study could represent an example for further studies where other influencing gastric motility hormones can be studied, such as gastrin, cholecystokinin, neurotensin and motilin.

## 5. Conclusions

TP is a high-metabolic impact procedure whose indications are limited to a few cases of advanced pancreatic cancer, IPMN, pancreatic metastasis or chronic pancreatitis. These patients are often affected by cachexia, malnutrition, sarcopenia and anorexia. Only a few articles in the recent literature have analyzed the nutritional aspects in TP or TPIAT.

Nutritional status is a prognostic factor both in TP for pancreatic cancer to support chemotherapy, prevent recurrence and prolong survival, and in TPIAT for chronic pancreatitis to improve postoperative glycemic control and to obtain better outcomes. PNI, sarcopenia, BMI and serum albumin levels reflect nutritional status.

The primary aim of parenteral or enteral nutrition in patient candidates to TP is to prevent or reduce cachexia. Enteral nutrition is always preferable to parenteral nutrition, and it also seems to be less expensive. However, sufficient oral feeding is often impossible to obtain within 7 days after surgery.

Nowadays, the nutritional consequences of TP, including diabetes control, are improved and become more manageable.

A considerable number of studies focused their attention on insulin and glucagon roles after TPIAT, but what emerged was that incretin hormones play a role after TPIAT, and this evidence can be the base for further future studies. Development of fatty liver and hepatic steatosis are two uncommon but worrisome findings after TP. A fatty pancreas may play a role in postoperative glycemic control after TPIAT.

The main limitations of this comprehensive review are the limited number and heterogeneity of the studies in recent literature regarding nutritional aspects in total pancreatectomy and the limited number of cases for each study.

Further researches are required to fully understand perioperative nutritional aspects in total pancreatectomy. Prospective randomized studies could be designed: (1) To investigate several pre-operative “diets”, identifying the optimal one which guarantees the best nutritional status for surgery; (2) To evaluate the post-operative impact of enteral nutrition compared to parenteral.

Furthermore, by exploiting post-processing imaging programs, pre-defined pre-operative parameters (e.g., the ratio between visceral and subcutaneous fat) might be investigated to identify groups of patients who are at a greater risk of nutritional deficiencies in the postoperative period. In fact, multicenter observational studies could be conducted to evaluate the correlation between pre-operative imaging parameters and the postoperative severity of nutritional deficiencies.

## Figures and Tables

**Table 1:** Selected articles.

Study	Year	Studt Design	Sample Size	Intervention Timing	Outcomes	Conclusion
Shi et al. [18]	2017	Single-centre	52 TP	Postoperative	Glycemic control Nutritional status	Improvement of glycemic control and nutritional status after TP is important to prevent early complications and tumor recurrence and to improve survival
Lundberg et al. [19]	2013	Single-centre	60 TPIAT	Preoperative chronic pancreatitis, stimulated insulin and C-peptide levels	Number of islet isolated	Normal stimulated C-peptide and fasting glucose correlate with low risk for low islet yield
Trikudanathan et al. [20]	2020	Single-centre	138 TPIAT (46 vs. 92)	Preoperative sarcopenia	Discharging to rehabilitation Islet yield LoS30-day readmission rate	Association between sarcopenia and an increased chance of discharge to a residential rehabilitation facility and with a poor islet yield during TPIAT
Takita et al. [21]	2011	Single-centre	12 TPIAT	BMI of pancreatic donor	Insulin independence Islet yield	Decreased C-peptide levels in the low-BMI group Better long-term graft function and islet yields in the high BMI group
Karagianni et al. [22]	2012	Review	11 studies	EN or PN postoperative support	Cachexia Toxicity to chemotherapy Nutritional status Reduced mortality (infectious complications, LoS) Postoperative gastric stasis	EN reduces gastrointestinal toxicity derived from chemotherapy Cyclic EN reduces postoperative gastrointestinal stasis Postoperative PN in every patient is not recommended
Andersen et al. [23]	2018	Single-centre	97 TP (57 vs. 40)	Postoperative parenteral nutrition vs. glucose infusion	Glycemic control Non-infectious post-operative complications	PN improves glycemic control and reduces non-infectious post-operative complications with respect to glucose infusion
Wagar et al. [24]	2017	Single-centre	67 TPIAT 44 vs. 23	Intraoperative goal-directed fluid therapy protocol vs. standard fluid therapy	Intraoperative complications (resuscitation, transfusion) Postoperative complications (graft function, 30-days complications)	Decreased intraoperative fluid resuscitation and blood transfusion using a goal-directed fluid therapy protocol vs. standard fluid Similar postoperative complications
Scholten et al. [25]	2019	Systematic review	21 studies TP 1536 pts	Functional outcome and quality of life after total pancreatectomy	QoL Endocrine insufficiency Exocrine insufficiency	QoL is affected adversely, in particular by the consider-able impact of diarrhea improvement in the management of diabetes after TP
Bogachus et al. [26]	2018	Single-centre	20 TPIAT (10 vs. 10)	Postoperative postprandial hypoglycemia	Decreases in postprandial glucose	Absent glucagon response contributes to postprandial hypoglycemia post-TPIAT
Hata et al. [27]	2016	Single-centre	43 TP	Postoperative development of hepatic steatosis	Relationship between postoperative hepatic steatosis and pancreatic insufficiency	Development of hepatic steatosis after TP is related to female sex and early nutritional status and prevented with high-dose pancreatic enzyme replacement therapy
Kizilgul et al. [28]	2018	Single-centre	79 TPIAT (53 vs. 26)	Preoperative pancreatic fat content	Insulin-dependent Postprandial glucose excursion	Intrapancreatic fat causes beta cell disfunction after TPIAT
McEachron et al. [29]	2020	Single-centre	34 TPIAT	Enteroendocrine postoperative changes	Stimulated levels of GLP-1, PYY and PP	GLP-1 and PYY levels are higher after TPIAT PP is not significantly lower after TPIAT

TP: total pancreatectomy; TPIAT: total pancreatectomy with islet auto-transplantation; LoS: length of hospital stay; BMI: body mass index; EN: enteral nutrition; PN: parenteral nutrition; QoL: quality of life; GLP-1: glucagon-like peptide 1; PYY: peptide YY; PP: Pancreatic polypeptide.

## References

[1] Casadei R., Monari F., Buscemi S., Laterza M., Ricci C., Rega D., D’Ambra M., Pezzilli R., Calculli L., Santini D. (2010). Total pancreatectomy: Indications, operative technique, and results: A single centre experience and review of literature. Updates Surg..

[2] Del Chiaro M., Rangelova E., Segersvärd R., Arnelo U. (2016). Are there still indications for total pancreatectomy?. Updates Surg..

[3] Di Franco G., Gianardi D., Palmeri M., Furbetta N., Guadagni S., Bianchini M., Bonari F., Sbrana A., Vasile E., Pollina L.E. (2020). Pancreatic resections for metastases: A twenty-year experience from a tertiary care center. Eur. J. Surg. Oncol..

[4] Di Franco G., Palmeri M., Sbrana A., Gianardi D., Furbetta N., Guadagni S., Bianchini M., Stefanini G., Adamo G., Pollina L.E. (2020). Renal cell carcinoma: The role of radical surgery on different patterns of local or distant recurrence. Surg. Oncol..

[5] Bozzetti F. (2009). Screening the nutritional status in oncology: A preliminary report on 1000 outpatients. Support. Care Cancer.

[6] Ockenga J., Valentini L. (2005). Review article: Anorexia and cachexia in gastrointestinal cancer. Aliment. Pharmacol. Ther..

[7] Di Luzio R., Moscatiello S., Marchesini G. (2010). Role of nutrition in gastrointestinal oncological patients. Eur. Rev. Med. Pharmacol. Sci..

[8] Min M., Patel B., Han S., Bocelli L., Kheder J., Vaze A., Wassef W. (2018). Exocrine pancreatic insufficiency and malnutrition in chronic pancreatitis: Identification, treatment, and consequences. Pancreas.

[9] Schnelldorfer T., Adams D.B. (2005). The effect of malnutrition on morbidity after Surgery for chronic pancreatitis. Am. Surg..

[10] Arvanitakis M., Ockenga J., Bezmarevic M., Gianotti L., Krznarić Ž., Lobo D.N., Löser C., Madl C., Meier R., Phillips M. (2020). ESPEN guideline on clinical nutrition in acute and chronic pancreatitis. Clin. Nutr..

[11] Wu Q., Zhang M., Qin Y., Jiang R., Chen H., Xu X., Yang T., Jiang K., Miao Y. (2015). Systematic review and meta-analysis of islet autotransplantation after total pancreatectomy in chronic pancreatitis patients. Endocr. J..

[12] Wahoff D.C., Papalois B.E., Najarian J.S., Kendall D.M., Farney A.C., Leone J.P., Jessurun J., Dunn D.L., Robertson R.P., Sutherland D.E.R. (1995). Autologous islet transplantation to prevent diabetes after pancreatic resection. Proceedings of the Annals of Surgery.

[13] Clayton H.A., Davies J.E., Pollard C.A., White S.A., Musto P.P., Dennison A.R. (2003). Pancreatectomy with islet autotransplantation for the treatment of severe chronic pancreatitis: The first 40 patients at the Leicester General Hospital. Transplantation.

[14] Hanazaki K., Maeda H., Okabayashi T. (2009). Relationship between perioperative glycemic control and postoperative infections. World J. Gastroenterol..

[15] Hackert T., Schütte K., Malfertheiner P. (2014). The pancreas: Causes for malabsorption. Visz. Gastrointest. Med. Surg..

[16] Higashiguchi T., Kita T., Noguchi T., Kawarada Y., Mizumoto R. (1988). Importance of nutritional management for the treatment of carcinoma of the pancreas. Gan To Kagaku Ryoho.

[17] Moher D., Liberati A., Tetzlaff J., Altman D.G., Altman D., Antes G., Atkins D., Barbour V., Barrowman N., Berlin J.A. (2009). Preferred reporting items for systematic reviews and meta-analyses: The PRISMA statement. Ann. Intern. Med..

[18] Shi H.J., Jin C., Fu D.L. (2017). Impact of postoperative glycemic control and nutritional status on clinical outcomes after total pancreatectomy. World J. Gastroenterol..

[19] Lundberg R., Beilman G.J., Dunn T.B., Pruett T.L., Chinnakotla S.C., Radosevich D.M., Robertson R.P., Ptacek P., Balamurugan A.N., Wilhelm J.J. (2013). Metabolic assessment prior to total pancreatectomy and islet autotransplant: Utility, limitations and potential. Am. J. Transplant..

[20] Trikudanathan G., Feussom G., Teigen L., Munigala S., Price K., Dirweesh A., Wilhelm J.J., Hering B.J., Kirchner V., Chinnakotla S. (2020). Pre-operative Sarcopenia Predicts Low Islet Cell Yield Following Total Pancreatectomy with Islet Autotransplantation for Chronic Pancreatitis. J. Gastrointest. Surg..

[21] Takita M., Naziruddin B., Matsumoto S., Noguchi H., Shimoda M., Chujo D., Itoh T., Sugimoto K., Tamura Y., Olsen G.S. (2011). Body mass index reflects islet isolation outcome in islet autotransplantation for patients with chronic pancreatitis. Cell Transplant..

[22] Karagianni V.T., Papalois A.E., Triantafillidis J.K. (2012). Nutritional Status and Nutritional Support Before and After Pancreatectomy for Pancreatic Cancer and Chronic Pancreatitis. Indian J. Surg. Oncol..

[23] Andersen S., Andersen A., Ringholm L., Hansen C.P., Storkholm J., Lillpers K., Schiøtz C., Mathiesen E.R. (2018). Parenteral nutrition and insulin per protocol improve diabetes management after total pancreatectomy. Dan. Med. J..

[24] Wagar M.K., Magnuson J., Liu P.T., Kirchner V., Wilhelm J.J., Freeman M.L., Bellin M.D., Pruett T.L., Beilman G.J., Dunn T.B. (2017). The impact of using an intraoperative goal directed fluid therapy protocol on clinical outcomes in patients undergoing total pancreatectomy and islet cell autotransplantation. Pancreatology.

[25] Scholten L., Stoop T.F., Del Chiaro M., Busch O.R., van Eijck C., Molenaar I.Q., de Vries J.H., Besselink M.G. (2019). Systematic review of functional outcome and quality of life after total pancreatectomy. Br. J. Surg..

[26] Bogachus L.D., Bellin M.D., Vella A., Paul Robertson R. (2018). Deficient Glucagon Response to Hypoglycemia during a Mixed Meal in Total Pancreatectomy/Islet Autotransplantation Recipients. J. Clin. Endocrinol. Metab..

[27] Hata T., Ishida M., Motoi F., Sakata N., Yoshimatsu G., Naitoh T., Katayose Y., Egawa S., Unno M. (2016). Clinical characteristics and risk factors for the development of postoperative hepatic steatosis after total pancreatectomy. Pancreas.

[28] Kizilgul M., Wilhelm J.J., Beilman G.J., Chinnakotla S., Dunn T.B., Pruett T.L., Abdulla M., Heller D., Freeman M.L., Schwarzenberg S.J. (2018). Effect of intrapancreatic fat on diabetes outcomes after total pancreatectomy with islet autotransplantation. J. Diabetes.

[29] McEachron K.R., Yang Y., Hodges J.S., Beilman G.J., Pruett T.L., Kirchner V.A., Dunn T.B., Freeman M.L., Trikudanathan G., Mulier K.E. (2020). Alterations in Enteroendocrine Hormones after Total Pancreatectomy with Islet Autotransplantation. Pancreas.

[30] Smale B.F., Mullen J.L., Buzby G.P., Rosato E.F. (1981). The efficacy of nutritional assessment and support in cancer surgery. Cancer.

[31] Onodera T., Goseki N., Kosaki G. (1984). Prognostic nutritional index in gastrointestinal surgery of malnourished cancer patients. Nippon Geka Gakkai Zasshi..

[32] Nozoe T., Kohno M., Iguchi T., Mori E., Maeda T., Matsukuma A., Ezaki T. (2012). The prognostic nutritional index can be a prognostic indicator in colorectal carcinoma. Surg. Today.

[33] Vashi P., Popiel B., Lammersfeld C., Gupta D. (2015). Outcomes of Systematic Nutritional Assessment and Medical Nutrition Therapy in Pancreatic Cancer. Pancreas.

[34] Geng Y., Qi Q., Sun M., Chen H., Wang P., Chen Z. (2015). Prognostic nutritional index predicts survival and correlates with systemic inflammatory response in advanced pancreatic cancer. Eur. J. Surg. Oncol..

[35] Kanda M., Fujii T., Kodera Y., Nagai S., Takeda S., Nakao A. (2011). Nutritional predictors of postoperative outcome in pancreatic cancer. Br. J. Surg..

[36] Schiesser M., Kirchhoff P., Müller M.K., Schäfer M., Clavien P.A. (2009). The correlation of nutrition risk index, nutrition risk score, and bioimpedance analysis with postoperative complications in patients undergoing gastrointestinal surgery. Surgery.

[37] Ikeguchi M., Goto K., Watanabe J., Urushibara S., Osaki T., Endo K., Tatebe S., Nakamura S. (2019). Clinical importance of preoperative and postoperative prognostic nutritional index in patients with pancreatic ductal adenocarcinoma. Ann. Hepato-Biliary-Pancreat. Surg..

[38] Nanashima A., Hiyoshi M., Imamura N., Yano K., Hamada T., Hamada R., Nagatomo K., Ikenoue M., Tobinaga S., Nagayasu T. (2019). Clinical significance of preoperative nutritional parameter and patient outcomes after pancreatectomy: A retrospective study at two academic institute. Ann. Hepato-Biliary-Pancreat. Surg..

[39] Chinnakotla S., Beilman G.J., Dunn T.B., Bellin M.D., Freeman M.L., Radosevich D.M., Arain M., Amateau S.K., Mallery J.S., Schwarzenberg S.J. (2015). Factors predicting outcomes after a total pancreatectomy and islet autotransplantation lessons learned from over 500 cases. Ann. Surg..

[40] Sutherland D.E.R., Radosevich D.M., Bellin M.D., Hering B.J., Beilman G.J., Dunn T.B., Chinnakotla S., Vickers S.M., Bland B., Balamurugan A.N. (2012). Total pancreatectomy and islet autotransplantation for chronic pancreatitis. J. Am. Coll. Surg..

[41] Khan K.M., Desai C.S., Kalb B., Patel C., Grigsby B.M., Jie T., Gruessner R.W.G., Rodriguez-Rilo H. (2013). MRI prediction of islet yield for autologous transplantation after total pancreatectomy for chronic pancreatitis. Dig. Dis. Sci..

[42] Bellin M.D., Freeman M.L., Schwarzenberg S.J., Dunn T.B., Beilman G.J., Vickers S.M., Chinnakotla S., Balamurugan A.N., Hering B.J., Radosevich D.M. (2011). Quality of Life Improves for Pediatric Patients After Total Pancreatectomy and Islet Autotransplant for Chronic Pancreatitis. Clin. Gastroenterol. Hepatol..

[43] Cruz-Jentoft A.J., Kiesswetter E., Drey M., Sieber C.C. (2017). Nutrition, frailty, and sarcopenia. Aging Clin. Exp. Res..

[44] Duggan S.N. (2017). Negotiating the complexities of exocrine and endocrine dysfunction in chronic pancreatitis. Proc. Nutr. Soc..

[45] Kuan L.L., Dennison A.R., Garcea G. (2021). Prevalence and Impact of Sarcopenia in Chronic Pancreatitis: A Review of the Literature. World J. Surg..

[46] Bassi C., Marchegiani G., Dervenis C., Sarr M., Abu Hilal M., Adham M., Allen P., Andersson R., Asbun H.J., Besselink M.G. (2017). The 2016 update of the International Study Group (ISGPS) definition and grading of postoperative pancreatic fistula: 11 Years After. Surgery.

[47] Olesen S.S., Büyükuslu A., Køhler M., Rasmussen H.H., Drewes A.M. (2019). Sarcopenia associates with increased hospitalization rates and reduced survival in patients with chronic pancreatitis. Pancreatology.

[48] La Torre M., Ziparo V., Nigri G., Cavallini M., Balducci G., Ramacciato G. (2013). Malnutrition and pancreatic surgery: Prevalence and outcomes. J. Surg. Oncol..

[49] Wehler M., Nichterlein R., Fischer B., Farnbacher M., Reulbach U., Hahn E.G., Schneider T. (2004). Factors associated with health-related quality of life in chronic pancreatitis. Am. J. Gastroenterol..

[50] Gruppo M., Angriman I., Martella B., Spolverato Y.C., Zingales F., Bardini R. (2018). Perioperative albumin ratio is associated with post-operative pancreatic fistula. ANZ J. Surg..

[51] Ryan A.M., Hearty A., Prichard R.S., Cunningham A., Rowley S.P., Reynolds J.V. (2007). Association of hypoalbuminemia on the first postoperative day and complications following esophagectomy. J. Gastrointest. Surg..

[52] Lohsiriwat V., Lohsiriwat D., Boonnuch W., Chinswangwatanakul V., Akaraviputh T., Lert-Akayamanee N. (2008). Pre-operative hypoalbuminemia is a major risk factor for postoperative complications following rectal cancer surgery. World J. Gastroenterol..

[53] Gibbs J., Cull W., Henderson W., Daley J., Hur K., Khuri S.F. (1999). Preoperative serum albumin level as a predictor of operative mortality and morbidity: Results from the National VA Surgical Risk Study. Arch. Surg..

[54] Billingsley K.G., Hur K., Henderson W.G., Daley J., Khuri S.F., Bell R.H. (2003). Outcome after pancreaticoduodenectomy for periampullary cancer: An analysis from the Veterans Affairs National Surgical Quality Improvement Program. Proceedings of the Journal of Gastrointestinal Surgery.

[55] Nakano Y., Kitago M., Shinoda M., Yagi H., Abe Y., Takano K., Oshima G., Takeuch A., Endo Y., Kitagawa Y. (2019). Prognostic significance of the postoperative level and recovery rate of serum albumin in patients with curatively resected pancreatic ductal adenocarcinoma. Mol. Clin. Oncol..

[56] Inui A. (1999). Cancer anorexia-cachexia syndrome: Are neuropeptides the key?. Cancer Res..

[57] Ramos E.J.B., Suzuki S., Marks D., Inui A., Asakawa A., Meguid M.M. (2004). Cancer anorexia-cachexia syndrome: Cytokines and neuropeptides. Curr. Opin. Clin. Nutr. Metab. Care.

[58] Gordon J.N., Trebble T.M., Ellis R.D., Duncan H.D., Johns T., Goggin P.M. (2005). Thalidomide in the treatment of cancer cachexia: A randomised placebo controlled trial. Gut.

[59] Brennan M.F., Pisters P.W.T., Posner M., Quesada O., Shike M. (1994). A prospective randomized trial of total parenteral nutrition after major pancreatic resection for malignancy. Ann. Surg..

[60] Ward N. (2003). Nutrition support to patients undergoing gastrointestinal surgery. Nutr. J..

[61] Liu Q., Liu Z., Chen H., Ma L., Liu L., Zhang J., He Y., Chen J., Qian Q. (2011). Treatment with growth hormone, somatostatin, and insulin in combination with hypocaloric parenteral nutrition in gastrointestinal cancer patients after surgery. Nutrition.

[62] Baradi H., Walsh R.M., Henderson J.M., Vogt D., Popovich M. (2004). Postoperative jejunal feeding and outcome of pancreaticoduodenectomy. In Proceedings of the Journal of Gastrointestinal Surgery. J. Gastrointest. Surg..

[63] Paccagnella A., Morassutti I., Rosti G. (2011). Nutritional intervention for improving treatment tolerance in cancer patients. Curr. Opin. Oncol..

[64] Barber M.D., Ross J.A., Voss A.C., Tisdale M.J., Fearon K.C.H. (1999). The effect of an oral nutritional supplement enriched with fish oil on weight-loss in patients with pancreatic cancer. Br. J. Cancer.

[65] Liu C., Du Z., Lou C., Wu C., Yuan Q., Wang J., Shu G., Wang Y. (2011). Enteral nutrition is superior to total parenteral nutrition for pancreatic cancer patients who underwent pancreaticoduodenectomy. Asia Pac. J. Clin. Nutr..

[66] Braga M., Gianotti L., Gentilini O., Parisi V., Salis C., Di Carlo V. (2001). Early postoperative enteral nutrition improves gut oxygenation and reduces costs compared with total parenteral nutrition. Crit. Care Med..

[67] Maskell C., Daniels P., Johnson C.D. (1999). Dietary intake after pancreatectomy. Br. J. Surg..

[68] Johnson P.M., Chen S.S., Santomango T.S., Williams P.E., Lacy D.B., McGuinness O.P. (2011). Continuous low-dose fructose infusion does not reverse glucagon-mediated decrease in hepatic glucose utilization. Metabolism.

[69] Yuan X.Y., Zhang C.H., He Y.L., Yuan Y.X., Cai S.R., Luo N.X., Zhan W.H., Cui J. (2008). Is albumin administration beneficial in early stage of postoperative hypoalbuminemia following gastrointestinal surgery?: A prospective randomized controlled trial. Am. J. Surg..

[70] Barbier L., Jamal W., Dokmak S., Aussilhou B., Corcos O., Ruszniewski P., Belghiti J., Sauvanet A. (2013). Impact of total pancreatectomy: Short- and long-term assessment. HPB.

[71] Roberts K.J., Blanco G., Webber J., Marudanayagam R., Sutcliffe R.P., Muiesan P., Bramhall S.R., Isaac J., Mirza D.F. (2014). How severe is diabetes after total pancreatectomy? A case-matched analysis. HPB.

[72] Tattersall R.B. (1997). Brittle diabetes revisited: The third Arnold Bloom memorial lecture. Diabet. Med..

[73] Butler P.C., Rizza R.A. (1989). Regulation of carbohydrate metabolism and response to hypoglycemia. Endocrinol. Metab. Clin. N. Am..

[74] Dresler C.M., Fortner J.G., McDermott K., Bajorunas D.R. (1991). Metabolic consequences of (regional) total pancreatectomy. Ann. Surg..

[75] Jamil L.H., Chindris A.M., Gill K.R.S., Scimeca D., Stauffer J.A., Heckman M.G., Meek S.E., Nguyen J.H., Asbun H.J., Raimondo M. (2012). Glycemic control after total pancreatectomy for intraductal papillary mucinous neoplasm: An exploratory study. HPB Surg..

[76] Jethwa P., Sodergren M., Lala A., Webber J., Buckels J.A.C., Bramhall S.R., Mirza D.F. (2006). Diabetic control after total pancreatectomy. Dig. Liver Dis..

[77] Wu W., Dodson R., Makary M.A., Weiss M.J., Hirose K., Cameron J.L., Ahuja N., Pawlik T.M., Wolfgang C.L., He J. (2016). A contemporary evaluation of the cause of death and long-term quality of life after total pancreatectomy. World J. Surg..

[78] Bellin M.D., Parazzoli S., Oseid E., Bogachus L.D., Schuetz C., Patti M.E., Dunn T., Pruett T., Balamurugan A.N., Hering B. (2014). Defective glucagon secretion during hypoglycemia after intrahepatic but not nonhepatic islet autotransplantation. Am. J. Transplant..

[79] Holst J.J., Holst Pedersen J., Baldissera F., Stadil F. (1983). Circulating glucagon after total pancreatectomy in man. Diabetologia.

[80] Yasui K. (1983). Effects of total pancreatectomy on the secretion of gut glucagon in humans. Jpn. J. Surg..

[81] Muller W.A., Brennan M.F., Tan M.H., Aoki T.T. (1974). Studies of glucagon secretion in pancreatectomized patients. Diabetes.

[82] Tanjoh K., Tomita R., Mera K., Hayashi N. (2002). Metabolic modulation by concomitant administration of insulin and glucagon in pancreatectomy patients. Hepatogastroenterology.

[83] Rodriguez-Diaz R., Caicedo A. (2013). Novel approaches to studying the role of innervation in the biology of pancreatic islets. Endocrinol. Metab. Clin. N. Am..

[84] Taborsky G.J., Mundinger T.O. (2012). Minireview: The role of the autonomic nervous system in mediating the glucagon response to hypoglycemia. Endocrinology.

[85] Petersen K.F., Price T.B., Bergeron R. (2004). Regulation of net hepatic glycogenolysis and gluconeogenesis during exercise: Impact of type 1 diabetes. J. Clin. Endocrinol. Metab..

[86] Bogachus L.D., Oseid E., Bellin M., Vella A., Robertson R.P. (2017). Deficient endogenous glucose production during exercise after total pancreatectomy/islet autotransplantation. J. Clin. Endocrinol. Metab..

[87] Bajorunas D.R., Fortner J.G., Jaspan J., Sherwin R.S. (1986). Total pancreatectomy increases the metabolic response to glucagon in humans. J. Clin. Endocrinol. Metab..

[88] Heptulla R.A., Rodriguez L.M., Bomgaars L., Raymond M.W. (2005). The role of amylin and glucagon in the dampening of glycemic excursions in children with type 1 diabetes. Diabetes.

[89] Heidt D.G., Burant C., Simeone D.M. (2007). Total pancreatectomy: Indications, operative technique, and postoperative sequelae. J. Gastrointest. Surg..

[90] Suzuki S., Miura J., Shimizu K., Tokushige K., Uchigata Y., Yamamoto M. (2016). Clinicophysiological outcomes after total pancreatectomy. Scand. J. Gastroenterol..

[91] Robertson R.P., Harmon J., Tran P.O.T., Poitout V. (2004). β-Cell Glucose Toxicity, Lipotoxicity, and Chronic Oxidative Stress in Type 2 Diabetes. Proceedings of the Diabetes.

[92] Pitt H.A. (2007). Hepato-pancreato-biliary fat: The good, the bad and the ugly. HPB.

[93] Cusi K. (2010). The role of adipose tissue and lipotoxicity in the pathogenesis of type 2 diabetes. Curr. Diab. Rep..

[94] Newsholme P., Keane D., Welters H.J., Morgan N.G. (2007). Life and death decisions of the pancreatic β-cell: The role of fatty acids. Clin. Sci..

[95] Al-Haddad M., Khashab M., Zyromski N., Pungpapong S., Wallace M.B., Scolapio J., Woodward T., Noh K., Raimondo M. (2009). Risk factors for hyperechogenic pancreas on endoscopic ultrasound: A case-control study. Pancreas.

[96] Wu W.C., Wang C.Y. (2013). Association between non-alcoholic fatty pancreatic disease (nafpd) and the metabolic syndrome: Case-control retrospective study. Cardiovasc. Diabetol..

[97] Harrison E., Lal S., McLaughlin J.T. (2013). Enteroendocrine cells in gastrointestinal pathophysiology. Curr. Opin. Pharmacol..

[98] Spreckley E., Murphy K.G. (2015). The L-Cell in nutritional sensing and the regulation of appetite. Front. Nutr..

[99] Beglinger C., Degen L. (2006). Gastrointestinal satiety signals in humans—Physiologic roles for GLP-1 and PYY ?. Physiol. Behav..

[100] Savage A.P., Adrian T.E., Carolan G., Chatterjee V.K., Bloom S.R. (1987). Effects of peptide YY (PYY) on mouth to caecum intestinal transit time and on the rate of gastric emptying in healthy volunteers. Gut.

[101] Nauck M.A., Niedereichholz U., Ettler R., Holst J.J., Ørskov C., Ritzel R., Schmiegel W.H. (1997). Glucagon-like peptide 1 inhibition of gastric emptying outweighs its insulinotropic effects in healthy humans. Am. J. Physiol. Endocrinol. Metab..

[102] Stanley S., Wynne K., Bloom S. (2004). Gastrointestinal Satiety Signals III. Glucagon-like peptide 1, oxyntomodulin, peptide YY, and pancreatic polypeptide. Am. J. Physiol. Gastrointest. Liver Physiol..

[103] Smits M.M., Tonneijck L., Muskiet M.H.A., Kramer M.H.H., Cahen D.L., van Raalte D.H. (2016). Gastrointestinal actions of glucagon-like peptide-1-based therapies: Glycaemic control beyond the pancreas. Diabetes Obes. Metab..

